# Comparative Effectiveness of Neoadjuvant Treatments for Resectable Gastroesophageal Cancer: A Network Meta-Analysis

**DOI:** 10.3389/fphar.2018.00872

**Published:** 2018-08-06

**Authors:** Zhaolun Cai, Yiqiong Yin, Zhou Zhao, Chunyu Xin, Zhaohui Cai, Yuan Yin, Chaoyong Shen, Xiaonan Yin, Jian Wang, Zhixin Chen, Ye Zhou, Bo Zhang

**Affiliations:** ^1^Department of Gastrointestinal Surgery, West China Hospital, Sichuan University, Chengdu, China; ^2^West China College of Public Health, Sichuan University, Chengdu, China; ^3^Department of Infectious Disease, Jiangsu Province Hospital of Traditional Chinese Medicine, Affiliated Hospital of Nanjing University of Chinese Medicine, Nanjing, China; ^4^The First College of Clinical Medicine, Nanjing University of Chinese Medicine, Nanjing, China; ^5^Department of Gastric Surgery, Fudan University Shanghai Cancer Center, Shanghai, China

**Keywords:** gastric cancer, neoadjuvant, chemotherapy, chemoradiotherapy, network meta-analysis

## Abstract

**Background:** Several neoadjuvant treatments are available for patients with resectable gastroesophageal cancer. We did a Bayesian network meta-analysis (NMA) to compare available treatments, summarizing the direct and indirect evidence.

**Method:** We searched relevant databases for randomized controlled trials of neoadjuvant treatments for resectable gastroesophageal cancer which compared two or more of the following treatments: surgery alone, perioperative docetaxel, oxaliplatin, leucovorin, and fluorouracil (FLOT), and neoadjuvant treatments listed in National Comprehensive Cancer Network guideline. Then we performed a NMA to summarize the direct and indirect evidence to estimate the relative efficacy for outcomes including overall survival (OS), progression-free survival and R0 resection rate. We calculated odds ratio (OR) and hazard ratio (HR) with 95% credible intervals (CrI) for dichotomous data and time-to-event data, respectively. We also calculated the surface under the cumulative ranking curve (SUCRA) value of each intervention to obtain a hierarchy of treatments.

**Result:** Eight eligible trials (2434 patients) were included in our NMA. The treatment with the highest probability of benefit on OS as compared with surgery alone was perioperative FLOT [HR = 0.58 with 95% CrI: (0.43, 0.78), SUCRA = 93%], followed by preoperative radiotherapy, paclitaxel, and carboplatin (RT/PC) [HR = 0.68 with 95% CrI: (0.53, 0.87), SUCRA = 72%], perioperative cisplatin with fluorouracil (CF) [HR = 0.70 with 95% CrI: (0.51, 0.95), SUCRA = 68%], and perioperative epirubicin, cisplatin, and fluorouracil or capecitabine (ECF/ECX) [HR = 0.75 with 95% CrI: (0.60, 0.94), SUCRA = 56%].

**Conclusion:** Compared with surgery alone, perioperative CF, perioperative ECF/ECX, perioperative FLOT, and preoperative RT/PC significantly improved survival. Perioperative FLOT is likely to be the most effective neoadjuvant treatment for the disease. Further clinical studies are needed and justified.

## Introduction

Gastric cancer (GC) is the third leading cause of cancer death worldwide in 2012 (Job Action Sheets, 2014). Complete surgical resection currently is the only curative treatment for localized GC (Van de Velde and Peeters, 2003; van Cutsem et al., 2011). However, GC is often diagnosed at an advanced stage which is unsuitable for radical surgery (Van de Velde and Peeters, 2003). Even despite potentially curative surgical resections, the prognosis of patients with more advanced GC (T2–4) remains poor due to metastatic disease or local recurrence after radical gastrectomy (Briasoulis et al., 2006; Reim et al., 2013). The high risk of disease relapse prompted the investigation of multidisciplinary strategies. Neoadjuvant chemotherapy or chemoradiotherapy with the advantages of downsizing the tumor before surgery and improving R0 resection rate (Xiong et al., 2014; Xu et al., 2014; Fu et al., 2015; Jiang et al., 2015; Miao et al., 2018) provides a therapeutic alternative for patients with resectable GC. Previous randomized controlled trials (RCTs) have assessed the outcomes of preoperative and perioperative treatments, producing conflicting results (Hartgrink et al., 2004; Burmeister et al., 2005; Cunningham et al., 2006; Shapiro et al., 2015). Efficacy of several neoadjuvant treatments has been established, and the National Comprehensive Cancer Network (NCCN) guideline for GC described numerous recommended neoadjuvant treatments (Ajani et al., 2016). Perioperative docetaxel, oxaliplatin, leucovorin, and fluorouracil (FLOT) have also demonstrated high efficacy against resectable gastroesophageal cancer (Al-Batran et al., 2017). However, the lack of head-to-head clinical trials made the role of optimal neoadjuvant treatments unknown.

To solve above problems, we conducted a Bayesian network meta-analysis (NMA). In the Bayesian hierarchical model, we can combine direct and indirect comparisons to compare two or more inventions at the same time when head-to-head studies are not available (Lumley, 2002; Song et al., 2003; Rucker, 2012; Dias et al., 2013). Our NMA aimed to summarize the direct and indirect evidence to obtain the estimates of relative effectiveness for perioperative FLOT and available neoadjuvant treatments listed in the NCCN guidelines for resectable GC.

## Materials and Methods

### Search Strategy

PubMed, Embase (Ovid), Cochrane Library (Ovid) were searched systematically for RCTs until the end of September 2017 without language restriction. We used combinations of the following terms: “gastric cancer,” “stomach neoplasms,” “stomach cancer,” “esophagogastric junction,” “gastroesophageal junction,” “neoadjuvant,” “adjuvant,” “perioperative,” “preoperative,” “chemotherapy,” “chemoradiotherapy,” “Randomized Controlled Trial,” “Controlled Clinical Trial” in accordance with the Cochrane Handbook (Higgins and Green, 2014).

The bibliographies of included studies were also checked for additional trials.

### Study Selection

We only included the RCTs that compared at least two arms of following treatments: surgery alone, perioperative FLOT, surgery combined with neoadjuvant treatments involving chemotherapy or chemoradiotherapy listed in the NCCN guidelines. Patients had been histologically proven gastric or lower third of the esophagus cancer with no evidence of distant metastasis.

We excluded studies if they were non-RCTs. Trials without enough data for us to estimate hazard ratios (HR) for survival were also excluded. Studies enrolling patients with esophageal cancer were excluded when data for gastric and lower third of the esophagus cancer were not separately extractable and/or the study included a limited number of patients with gastroesophageal cancer (<80%).

### Assessment of Risk of Bias and Data Collection

Qualitative assessment and data extraction were finished by two investigators independently; disagreements were resolved in discussion with a third author. The two authors independently extracted data from each enrolled study according to the same standardized collection form.

We collected the data regarding study quality, the year of publication, country, the sample size, treatment strategies, tumor site, median follow-up time, and outcomes.

The primary outcome was overall survival (OS). Secondary outcomes were progression-free survival (PFS) and R0 resection rate. We used HR and 95% Confidence Interval (CI) to assess OS and PFS.

The quality and the risk of bias of RCTs were assessed by Cochrane Collaboration’s tool in terms of sequence generation, allocation concealment, blinding of participants and researchers, incomplete outcome data, selective reporting, and other bias (Higgins et al., 2011; Higgins and Green, 2014). Blinding was not performed in all the enrolled trials, but assessment of survival was not likely to be influenced by lack of blinding of participants and researchers.

### Statistical Analysis

The primary outcome of our NMA was OS, and the secondary outcomes were PFS and R0 resection rate. Time-to-event outcomes such as OS and PFS were assessed by HRs which take the number and timing of events into consideration with its 95% CI (Higgins and Green, 2014). For dichotomous data, treatment effects were expressed as odds ratio (OR). 95% credible intervals (CrIs) which could be interpreted as 95% CI were used for the estimating treatment effects of NMA.

The NMA was performed in R, version 3.3.3 and Stata, version 12 (StataCorp., College Station, TX, United States) with the Bayesian methods (Lumley, 2002) and fixed-effect model. We used the node-split method to assess inconsistency between indirect and direct comparisons in closed loops, and a *P*-value lower than 0.05 indicates a statistically significant inconsistency (Dias et al., 2010). The consistency model was used when there was no significant inconsistency; otherwise, the inconsistency model was conducted.

We calculated values of the surface under the cumulative ranking curve (SUCRA) of each intervention to obtain a hierarchy of treatments. A higher SUCRA value indicated a better efficacy (Salanti et al., 2011).

## Results

### Study Selection and Characteristics

**FIGURE 1:**
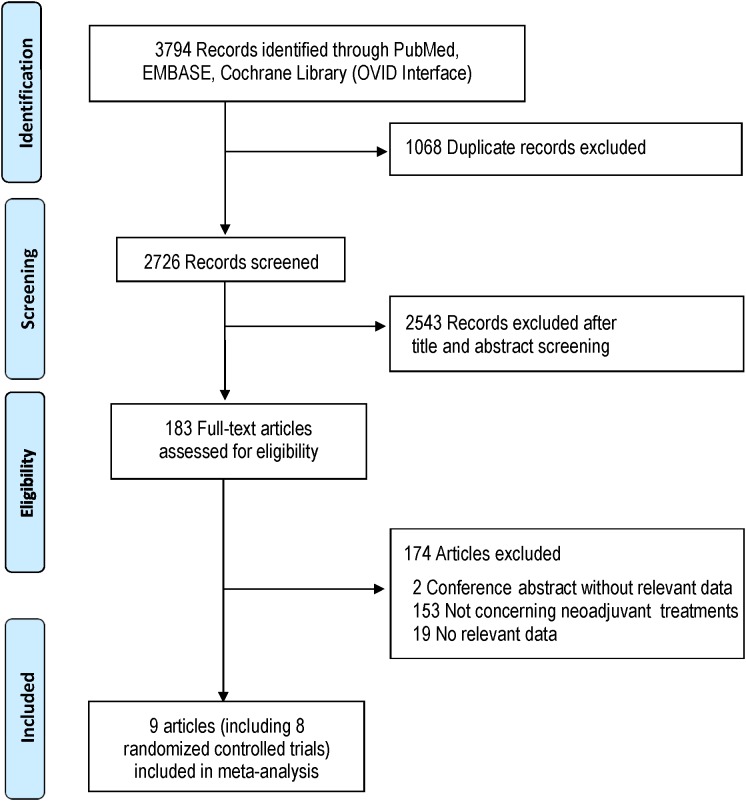
Study flow diagram.

The systematic search identified 3794 potentially relevant studies. Eight studies which met the inclusion criteria were included for the study (Burmeister et al., 2005; Cunningham et al., 2006; Stahl et al., 2009; Schuhmacher et al., 2010; Ychou et al., 2011; Shapiro et al., 2015; Klevebro et al., 2016; Al-Batran et al., 2017). Trials reported by Burmeister et al. (2005) and Klevebro et al. (2016) included several subgroups, and the subset with gastric or lower third of the esophagus cancer was used for the NMA. Literatures screening process was shown in **Figure 1** in accordance with PRISMA flowchart (Moher et al., 2009).

**FIGURE 2:**
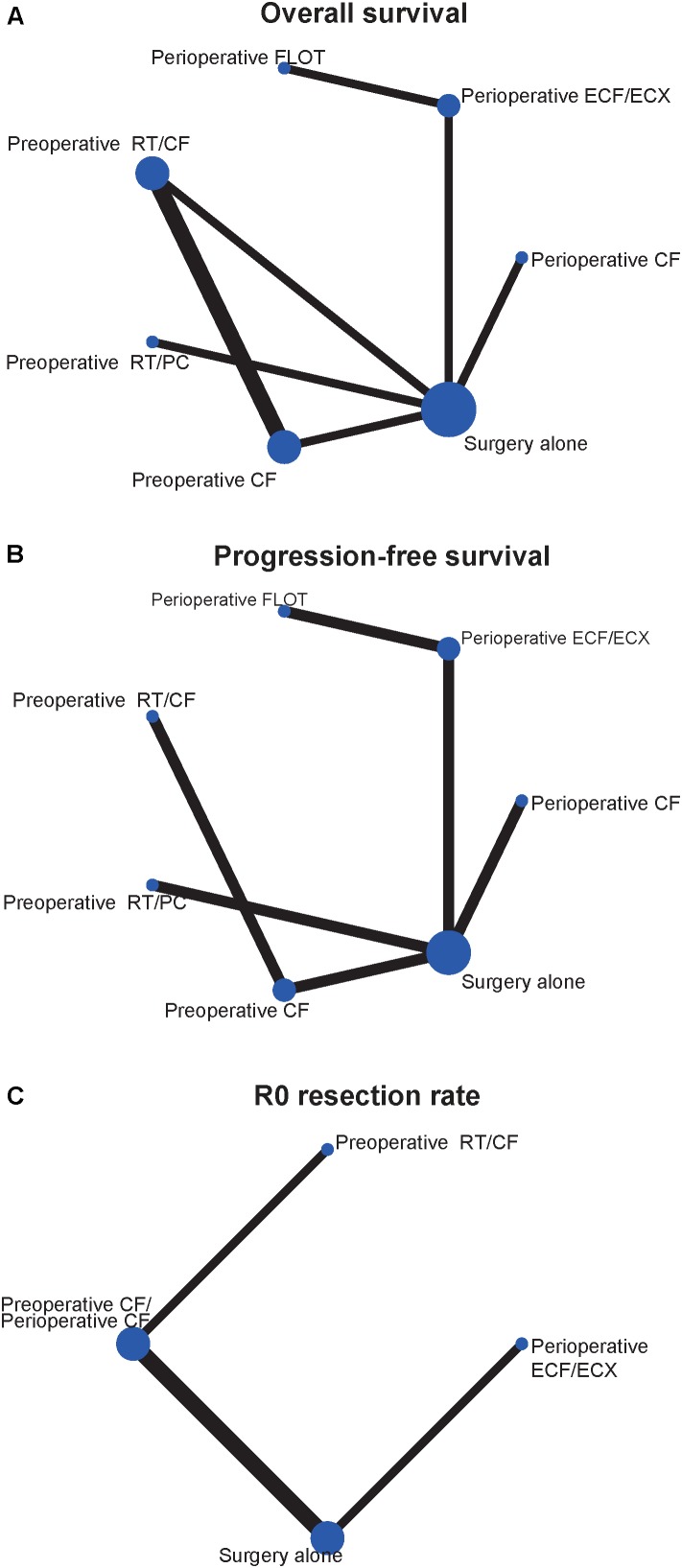
Network of the comparisons for the Bayesian network meta-analysis. **(A)** Overall survival; **(B)** progression-free survival; **(C)** R0 resection rate. The size of the nodes and the thickness of the edges are weighted according to the number of studies evaluating each treatment and direct comparison, respectively.

The characteristics of included studies are shown in **Table 1**. A total of 2434 patient treated in seven different treatments were included: 701 treated with surgery alone; 113, perioperative cisplatin with fluorouracil (CF); 207, preoperative CF; 610, perioperative epirubicin, cisplatin, and fluorouracil or capecitabine (ECF/ECX); 356, perioperative FLOT; 234, preoperative radiotherapy combined with CF (RT/CF); 213, preoperative radiotherapy, paclitaxel, and carboplatin (RT/PC). The network plots of OS, PFS, and R0 resection were shown in **Figure 2**, each node represents a treatment and the thickness of lines represents the number of trials.

**Table 1:** Study and patient population characteristics of included studies.

Author	Year	Country	Sample size (intervention/control)	Intervention		Control		Tumor site (%)	Median follow-up (months)
				Preoperative	Postoperative	Preoperative	Postoperative		
Ychou et al.	2011	France	113/111	5-FU Cisplatin	5-FU Cisplatin	–	–	Stomach (24%) GOJ (64%) Lower esophagus (11%)	68.4
Shapiro et al.	2014	Netherlands	213/161	Carboplatin Paclitaxel Radiotherapy	–	–	–	GOJ (24%) Lower esophagus (58%) Others (18%)	84.1
Schuhmacher et al.	2010	Europe	72/72	5-FU Cisplatin	–	–	–	Stomach (NR) GOJ (NR)	52.8
Al-Batran et al.	2017	Germany	360/356	Epirubicin Cisplatin 5-FU/CAP	Epirubicin Cisplatin 5-FU/CAP	5-FU Leucovorin Oxaliplatin Docetaxel	5-FU Leucovorin Oxaliplatin Docetaxel	Stomach (43%) GOJ (56%)	43
Klevebro et al.	2016	Sweden	75/76	5-FU Cisplatin Radiotherapy	–	5-FU Cisplatin	–	GOJ (17%) Lower esophagus (66%)	NR
Cunningham, D., et al.	2006	UK	250/253	5-FU Cisplatin Epirubicin	5-FU Cisplatin Epirubicin	–	–	Stomach (75%) GOJ (10%) Lower esophagus (15%)	49
Stahl et al.	2017	Germany	60/59	5-FU Cisplatin Radiotherapy	–	5-FU Cisplatin	–	Stomach and GOJ (45%) Lower esophagus (55%)	126.5
Burmeister et al.	2005	Australia	99/104	5-FU Cisplatin Radiotherapy	–	–	–	Lower esophagus (100%)	65

*NA, not available; NR, not reported; 5-FU, fluorouracil; CAP, capecitabine; GOJ, gastroesophageal junction.*

### Network Meta-Analysis

#### Overall Survival

**FIGURE 3:**
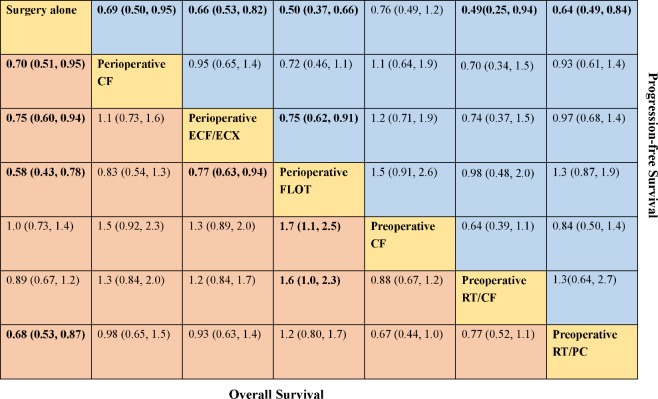
Comparative effectiveness of neoadjuvant treatments in network meta-analysis. Hazard ratio (95% credible interval) for comparisons are in cells in common between column-defining and row-defining treatment. Bold cells are significant. For overall survival, hazard ratio <1 favors row-defining treatment. For progression-free survival, hazard ratio <1 favors column-defining treatment.

**FIGURE 4:**
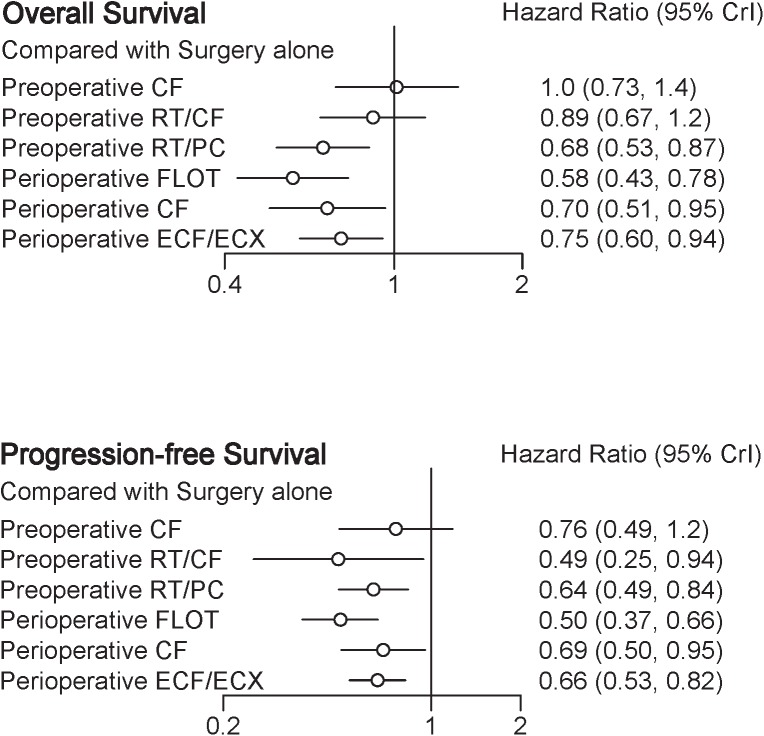
Forest plots of results with surgery alone as the common reference.

A total of eight trials contributed to our analysis, comparing the seven treatments. HRs were explicitly reported in all the eight trials. We summarize the comparisons analyzed by the Bayesian NMA in **Figure 3** and present the forest plots of meta-analysis comparing neoadjuvant treatments with surgery alone in **Figure 4**. Four treatments which significantly improved prognosis as compared with surgery alone were perioperative CF [HR = 0.70 with 95% CrI: (0.51, 0.95)], perioperative ECF/ECX [HR = 0.75 with 95% CrI: (0.60, 0.94)], perioperative FLOT [HR = 0.58 with 95% CrI: (0.43, 0.78)], and preoperative RT/PC [HR = 0.68 with 95% CrI: (0.53, 0.87)]. The rest of enrolled neoadjuvant treatments were not associated with an improved OS when compared with surgery alone. Adding radiation to preoperative CF also provided little further survival benefit as compared with preoperative CF [HR = 0.88 with 95% CrI: (0.67, 1.2)].

Perioperative FLOT had a statistical advantage over perioperative ECF/ECX, preoperative CT, and preoperative RT/CF in OS and showed a statistically non-significant trend to better survival as compared with the rest of treatments. No significant difference reached between the rest of four treatments which significantly improved OS as compared with surgery alone. The SUCRA values indicated that perioperative FLOT had the highest probability of being the best treatment for OS (SUCRA = 93%), followed by preoperative RT/PC (SUCRA = 72%), perioperative CF (SUCRA = 68%), and perioperative ECF/ECX (SUCRA = 56%). Surgery alone had the least chance of improving OS (SUCRA = 12%) (**Table 2**).

**Table 2:** The SUCRA value of different treatments on each outcome.

Treatments	Perioperative CF	Perioperative ECF/ECX	Perioperative FLOT	Preoperative CF	Preoperative RT/CF	Preoperative RT/PC	Surgery alone
OS	0.68	0.56	0.93	0.16	0.32	0.72	0.12
PFS	0.44	0.48	0.87	0.31	0.81	0.54	0.02

*A higher SUCRA value indicates a better efficacy.*

#### Progression-Free Survival

In terms of PFS, seven treatments were compared, and six trials contributed to the analysis (Cunningham et al., 2006; Schuhmacher et al., 2010; Ychou et al., 2011; Shapiro et al., 2015; Al-Batran et al., 2017; Stahl et al., 2017). HRs were explicitly reported in all the six trials. In **Figure 3** we summarize the comparisons analyzed by the Bayesian methods. The forest plots of results with surgery alone as the common reference are illustrated in **Figure 4**. Five treatments which reached statistical significance in terms of PFS as compared with surgery alone were perioperative FLOT [HR = 0.50 with 95% CrI: (0.37, 0.66)], preoperative RT/CF [HR = 0.49 with 95% CrI: (0.25, 0.94)], preoperative RT/PC [HR = 0.64 with 95% CrI: (0.49, 0.84)], perioperative ECF/ECX [HR = 0.66 with 95% CrI: (0.53, 0.82)], and perioperative CF [HR = 0.69 with 95% CrI: (0.50, 0.95)]. Preoperative CF alone showed no statistically significant survival benefit when compared with surgery alone [HR = 0.76 with 95% CrI: (0.49, 1.2)].

Perioperative FLOT also had a statistical advantage over perioperative ECF/ECX in PFS and showed a statistically non-significant trend to better survival as compared with the rest of treatments. No significant difference reached between the rest of four treatments which significantly improved PFS as compared with surgery alone. SUCRA values indicated that perioperative FLOT had the highest probability of being the best treatment for PFS (SUCRA = 87%), followed by preoperative RT/CF (SUCRA = 81%), preoperative RT/PC (SUCRA = 54%), perioperative ECF/ECX (SUCRA = 48%), and perioperative CF (SUCRA = 44%). Surgery alone had the least chance of improving PFS (**Table 2**).

#### R0 Resection Rate

Four trials (Cunningham et al., 2006; Stahl et al., 2009; Schuhmacher et al., 2010; Ychou et al., 2011) contributed to our analysis of R0 resection rate, comparing the three preoperative treatments. Perioperative/Preoperative CF was shown to have a significantly increased curative resection rate compared with surgery alone group [OR = 2 with 95% CrI: (1.2, 3.4)]. Preoperative RT/CF showed a trend to better resection rate as compared surgery alone [OR = 2.3 with 95% CrI: (0.88, 5.9)]. Perioperative ECF/ECX did not significantly improve R0 resection rate as compared with surgery alone [OR = 1.1 with 95% CrI: (0.78, 1.7)]. Perioperative/Preoperative CF also showed a statistically non-significant trend to better R0 resection rate as compared with perioperative ECF/ECX [OR = 1.8 with 95% CrI: (0.95, 3.4)].

### Quality of Evidence

**FIGURE 5:**
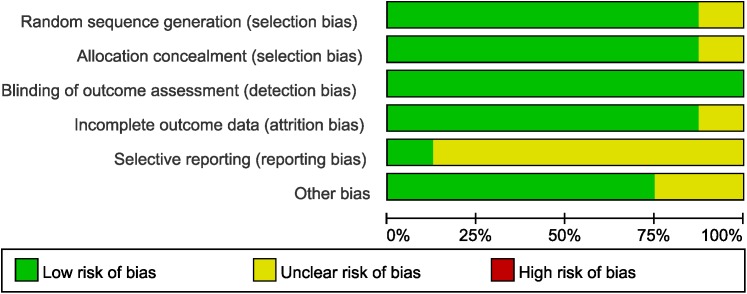
Risk of bias graph for all studies included.

The assessment of the risk of bias for selected studies in the NMA was shown in **Figure 5** according to the Cochrane risk-of-bias tool, indicating low risk of bias.

The node-splitting method suggested that there was no statistically significant inconsistency in any closed loop (*P* > 0.05) (Supplementary Figure S1).

## Discussion

In the absence of head-to-head comparative data, a definitive statement on the optimum neoadjuvant treatment for resectable gastroesophageal cancer is still absent. NMA synthesizing both direct and indirect evidence provides more powerful estimates and allows comparisons of relative effectiveness between interventions that have never been compared head to head. In this NMA, we combined direct and indirect evidence from seven RCTs involving 2434 patients with resectable gastroesophageal cancer to estimate the relative efficacy of included neoadjuvant treatments for outcomes including OS, PFS, and R0 resection rate.

We made several key observations regarding OS: (1) perioperative CF, perioperative ECF/ECX, perioperative FLOT, and preoperative RT/PC were superior to surgery alone for improving OS; (2) SUCRA value for indirect comparison indicated that perioperative FLOT is more likely to be the most effective treatment. Perioperative FLOT also showed a trend to better survival as compared with the rest of treatments, and the statistically significant difference was reached for the comparisons of perioperative FLOT versus perioperative ECF/ECX, preoperative CT, and preoperative RT/CF. (3) Preoperative CF and preoperative RT/CF failed to detect survival benefits as compared with surgical resection alone, which might result from inadequate statistical power due to the low number of events in relevant groups or the real possibility that preoperative CF or preoperative RT/CF does not have a beneficial impact on patients with the disease (Schuhmacher et al., 2010). Besides, no statistical difference was reached between preoperative CF and preoperative RT/CF. The previous study indicated a survival advantage for preoperative chemoradiotherapy compared with preoperative chemotherapy in adenocarcinomas of the esophagogastric junction, however, failed to achieve statistical significance. On the other hand, a meta-analysis reported the survival advantage from preoperative chemoradiotherapy over preoperative chemotherapy (Fu et al., 2015). Thus, the effect of adding radiotherapy to preoperative chemotherapy separately is still uncertain, and more high-quality RCTs are needed; (4) preoperative CF failed to demonstrate a survival benefit. Perioperative CF, however, improved survival significantly. Among preoperative, postoperative, and perioperative treatments, which is the key to improve survival is still controversial. Recently a trial has been conducted to investigate effects of postoperative oxaliplatin combined with S-1 and oxaliplatin with capecitabine, and perioperative oxaliplatin and S-1 on locally advanced GC. The patient recruitment had finished, and disease-free survival would be achieved in 2 years (Ji et al., 2017). The results would provide the clues to the question mentioned above.

The JCOG0501 trial explored neoadjuvant S-1 and cisplatin versus surgical resection followed by S-1 for type 4 and large type 3 GC, and the results suggested that neoadjuvant chemotherapy was safely performed without increasing morbidity and mortality (Japan Clinical Oncology Group, 2005). The results of survival will be demonstrated soon and promising.

When we were focusing on the results of PFS, five treatments which were shown to have a significantly improved prognosis as compared with surgery alone were perioperative CF, perioperative ECF/ECX, perioperative FLOT, preoperative RT/CF, and preoperative RT/PC. The results demonstrated perioperative FLOT might be the best treatment. No statistically significant difference reached between the rest treatments which improved the PFS.

Our analysis of R0 resection rate showed best results with perioperative/preoperative CF. Perioperative ECF/ECX failed to improve R0 resection rate. It is worth mentioning that preoperative CF was associated with a higher complete resection rate, however, failed to demonstrate a statistically significant survival benefit. Perioperative ECF/ECX which did not improve R0 resection rate, however, significantly improved OS and PFS. Whether improved R0 resection rate is associated with a better survival merits further discussion.

Our NMA has several limitations. First, based on meta-analyses of summary data, it was difficult for us to explore the impact of tumor location which might be the potential source of heterogeneity. Second, survival benefits must be balanced against risk of adverse effects. However, we could not conduct a quantitative synthesis on adverse effects associated with the relevant treatments because the data on adverse events were rarely available. Al-Batran et al. (2016) suggested that non-surgical adverse events in both perioperative ECF/ECX and perioperative FLOT were well tolerated and the incidences of adverse events were similar, and further clinical studies are needed. Third, some additional neoadjuvant treatments including S-1 combined with cisplatin and paclitaxel plus cisplatin are under study for the treatment of resectable GC (Yoshikawa et al., 2016). The study suggested that two courses of S-1 and cisplatin should be recommended as a reference arm in future to evaluate neoadjuvant chemotherapy for GC (Yoshikawa et al., 2016). However, the treatments mentioned above were excluded for the shortage of treatments which could connect the network nodes. Fourth, only a limited number of studies (*n* = 8) were included in the analysis. Furthermore, the best performing treatment (perioperative FLOT) has been investigated in only one study. Although no obvious inconsistency and severe risk of bias were detected, the limited number of trials and patients weakens the external validity of our study.

## Conclusion

The NMA provides the first comparison between neoadjuvant treatments for resectable gastroesophageal cancer. In the absence of head to head clinical trials to guide the choice of treatment, it has been unclear which treatment is optimal. The results show that OS is improved with perioperative CF, perioperative ECF/ECX, perioperative FLOT, and preoperative RT/PC. Perioperative FLOT is likely to be the most effective neoadjuvant treatment for the disease. Still, large prospective studies are required to investigate the optimal neoadjuvant treatment for the disease.

## Author Contributions

BZ, ZlC, and YiY designed the study. JW and XY screened studies and extracted data. Disagreements were resolved by discussion with YuY. ZlC and ZZ performed the statistical analyses. CX and ZhC prepared the figures and tables. BZ, ZxC, YZ, CS, and ZhC reviewed the results, interpreted data, and wrote the manuscript. All authors read and approved the final version of the paper.

## Conflict of Interest Statement

The authors declare that the research was conducted in the absence of any commercial or financial relationships that could be construed as a potential conflict of interest.
