# Supplementary material for: Comparative Effectiveness of Neoadjuvant Treatments for Resectable Gastroesophageal Cancer: A Network Meta-Analysis
Source: Front Pharmacol. 2018 Aug 6;9:872. doi: 10.3389/fphar.2018.00872 (PMC6087755; doi:10.3389/fphar.2018.00872)
Supplement: Supplementary file 1 [file Data_Sheet_1.docx]

**SUPPLEMENTARY MATERIAL**

**Comparative effectiveness of neoadjuvant treatments for resectable gastroesophageal cancer: a network meta-analysis**

Zhaolun Cai^1^* · Yiqiong Yin^1^* · Zhou Zhao^1^ · Chunyu Xin^3^ · Zhaohui Cai^4^ · Yuan Yin^1^ · Chaoyong Shen^1^· Xiaonan Yin^1^· Jian Wang^1^· Zhixin Chen^1^ · Ye Zhou^2^ · Bo Zhang^1Δ^

1. Department of Gastrointestinal Surgery, West China Hospital, Sichuan University, Chengdu 610041, Sichuan, China.

2. Department of Gastric Surgery, Fudan University Shanghai Cancer Center, Shanghai 200032, China.

3. West China College of Public Health, Sichuan University, Chengdu 610041, Sichuan, China.

4. Jiangsu Province Hospital of TCM, Affiliated Hospital of Nanjing University of TCM, Nanjing, China.

* These authors contributed equally to the research.

**Δ Corresponding Author** Bo Zhang, PhD, Department of Gastrointestinal Surgery, West China Hospital, Sichuan University, Chengdu 610041, Sichuan, China. Telephone number: +86-18980601891; Fax number: +86-28-85422872; E-mail address: hxwcwk@126.com.

**FIGURE S1**. Comparison between direct and indirect evidence for overall survival
